# Chirality-biased protein expression profile during early stages of bone regeneration

**DOI:** 10.3389/fbioe.2023.1217919

**Published:** 2023-07-18

**Authors:** Qiang Zeng, Huimin Zheng, Boon Chin Heng, Weitong Yao, Yue Yang, Shengjie Jiang, Xuliang Deng

**Affiliations:** ^1^ Institute of Medical Technology, Peking University Health Science Center, Beijing, China; ^2^ Beijing Laboratory of Biomedical Materials, Department of Geriatric Dentistry, Peking University School and Hospital of Stomatology, Beijing, China; ^3^ Hubei Jiangxia Laboratory, Wuhan, China; ^4^ Department of Prosthodontics, The First Clinical Division, Peking University School and Hospital of Stomatology, Beijing, China

**Keywords:** chiral matrix, differentially-expressed proteins, protein-protein interaction, macrophages, bone regeneration

## Abstract

**Introduction:** Chirality is a crucial mechanical cue within the extracellular matrix during tissue repair and regeneration. Despite its key roles in cell behavior and regeneration efficacy, our understanding of chirality-biased protein profile in vivo remains unclear.

**Methods:** In this study, we characterized the proteomic profile of proteins extracted from bone defect areas implanted with left-handed and right-handed scaffold matrices during the early healing stage. We identified differentially-expressed proteins between the two groups and detected heterogenic characteristic signatures on day 3 and day 7 time points.

**Results:** Proteomic analysis showed that left-handed chirality could upregulate cell adhesion-related and GTPase-related proteins on day 3 and day 7. Besides, interaction analysis and in vitro verification results indicated that the left-handed chiral scaffold material activated Rho GTPase and Akt1, ultimately leading to M2 polarization of macrophages.

**Discussion:** In summary, our study thus improved understanding of the regenerative processes facilitated by chiral materials by characterizing the protein atlas in the context of bone defect repair and exploring the underlying molecular mechanisms of chirality-mediated polarization differences in macrophages.

## 1 Introduction

The immune response plays a fundamental role in repairing tissue defects. The appropriate polarization of macrophages is critical for initiating tissue regeneration in bone defect areas (Willenborg and Eming, 2018). During the initial stages of bone healing, pro-inflammatory M1 macrophages eliminate cellular debris and apoptotic cells via phagocytosis, while anti-inflammatory M2 macrophages play a crucial role in promoting cell proliferation and tissue repair by secreting chemokines and recruiting stem cells to the defect area (Whitaker et al., 2021). Imbalances between the M1 and M2 macrophage subtypes can lead to slower tissue repair rates and lower repair quality in clinical settings. Therefore, considering the effects of immune regulatory factors on maintaining tissue stability is of key significance in biomaterial design.

Various physical and chemical cues of biomaterials, such as stiffness, mechanical loading, charge, wettability and pH, can regulate regeneration efficiency by affecting the polarization state of macrophages (Anselmo et al., 2015; Solis et al., 2019; Zarubova et al., 2022). Among all the cues of microenvironment, left-handed chirality has been reported to promote the proliferation and adhesion of cells, resulting in improved effect of osteogenic differentiation, periodontal tissue regeneration and wound healing (Zhao et al., 2017; Griffin et al., 2021; Zhang et al., 2021; Shi et al., 2022). Besides, left-hand chiral matrix can activate macrophages through fibronectin recognition, thereby polarizing macrophages towards the M2 subtype via cell adhesion signaling pathways (Jiang et al., 2022). However, the effect of chiral microenvironment on proteome *in vivo* and its effect on macrophages during bone defect repair have not yet been explored.

In this study, we developed a rat cranial bone defect model in which was implanted left-handed and right-handed chiral matrices. Tissue fluids were collected from the bone defect areas on both day 3 and day 7 post-implantation in order to analyze the proteomic characteristics of the samples. Our findings indicated that differentially-expressed proteins (DEPs) undergo a time-course shift during the initial stages of bone repair. Regeneration and adhesion proteins were upregulated in left-handed group. Further *in vitro* validation assays were performed to identify key role of Akt1 involved in this shift. Proteomic analysis was used to help evaluate the efficacy of chiral materials and further guide the mechanism investigation of biomaterials affected regeneration. Ultimately, our study produced a comprehensive atlas of proteins that are affected by matrix chirality within the bone defect area during the early stages of healing.

## 2 Materials and methods

### 2.1 Chiral matrix synthesis and preparation

Two chiral matrices were synthesized according to our previous study (Jiang et al., 2022). The chiral material p-Ph-(D- Phe-NHCH_2_CH_2_OCH_2_CH_2_OH)_2_ was named D and p-Ph-(L- Phe-NHCH_2_CH_2_OCH_2_CH_2_OH)_2_ was named L. Chiral matrices used for *in vivo* implantation were prepared 1 day in advance. DMEM (Cyagen Biosciences, Inc.) were mixed into concentrated solution of the gelator in DMSO. After the chiral matrices were formed, DMEM were added to keep the matrices moist.

### 2.2 Rat cranial bone defect model *in vivo*

The cranial bone defect model was created in healthy 5-week old SD rats (Wang et al., 2022; Guo and He, 2023). All protocols and procedures are compliant with the guidelines prescribed by the Association for Assessment and Accreditation of Laboratory Animal Care, approved by the Peking University Health Centre Institutional Animal Care and Use Committee and Peking University Health Centre Ethics Committee (LA2019320). Each group was comprised of five rats. The rats were anesthetized with 1% (w/v) sodium pentobarbital solution (40 mg/kg). After disinfection and incision, a critically-sized defect of 5 mm was created on the rat crania by a trephine bar. The periosteum was completely removed. The freshly-formed cranial defects were then injected with pre-formed chiral matrices. The total injection volume for each rat was maintained at 100 μL. Then non-resorbable membranes were used to guide tissue regeneration. Penicillin was administered to prevent infection following surgery. Three days and 7 days after the surgery, the rats were euthanized using CO_2_ asphyxiation, and tissue fluids from the defect area were collected. Protease inhibitor was added at 10% (v/v) of the lysate, and after centrifuging the mixture at 14,100 × g for 30 min, the supernatant was collected for LC-MS/MS analysis.

### 2.3 LC-MS/MS analysis

The analysis of tryptic peptides was conducted using nanoflow LC-MS/MS technology, which involved the use of a quadrupole Orbitrap mass spectrometer (Q Exactive HF-X, Thermo Fisher Scientific, Bremen, Germany) coupled with an EASY nLC 1,200 ultra-high pressure system (Thermo Fisher Scientific) via a nano-electrospray ion source. Five hundred ng of peptides were loaded onto a 25 cm column (150 μm inner diameter), which was packed with ReproSil-Pur C18-AQ 1.9-µm silica beads (Beijing Qinglian Biotech Co., Ltd., Beijing, China). Solvent A was composed of 0.1% (v/v) formic acid in water, and solvent B was composed of 80% (v/v) ACN and 0.1% (v/v) formic acid in water. Peptides were separated using a gradient from 8% to 12% B in 5 min, then 12%–30% B in 33 min, followed by a step up to 40% in 7 min, and a 15 min wash at 95% B at a flow rate of 600 nL per minute. The total duration of the run was 60 min, during which time the column was kept at a temperature of 60°C using an in-house-developed oven. The mass spectrometer was operated in “top-40” data-dependent mode, wherein the Orbitrap mass analyzer (120,000 resolutions, 350–1,500 m/z range) was used to collect MS spectra, with an automatic gain control (AGC) target of 3E6 and a maximum ion injection time of 80 m. The most intense ions from the full scan were isolated with an isolation width of 1.6 m/z, following higher-energy collisional dissociation (HCD) with a normalized collision energy (NCE) of 27, MS/MS spectra were collected in the Orbitrap (15,000 resolution) with an AGC target of 5E4 and a maximum ion injection time of 45 m. Precursor dynamic exclusion was enabled for 16 s.

### 2.4 Data analysis

#### 2.4.1 The identification and quantitation of proteins

All RAW files underwent analysis using the Proteome Discoverer suite (version 2.4 from Thermo Fisher Scientific). Then, MS2 spectra were compared against the UniProtKB human proteome database which contained both Swiss-Prot and TrEMBL human reference protein sequences, a total of 20,373 target sequences downloaded on 17 March 2022. The Sequest HT search engine was employed, and the following parameters were set: full tryptic specificity, maximum of two missed cleavages, minimum peptide length of 6 amino acids, and fixed carbamidomethylation of cysteine residues. Variable modifications for oxidized methionine residues were also included, and the precursor mass tolerance was set at 15 ppm with a fragment mass tolerance of 0.02 Da for MS2 spectra collected in the Orbitrap. Furthermore, a percolator was utilized to filter peptide spectral matches and peptides to a false discovery rate (FDR) of less than 1%. After spectral assignment was complete, peptides were assembled into proteins and were further filtered based on the combined probabilities of their constituent peptides to a final FDR of 1%. An important aspect of this analysis is that it only considered unique and razor peptides for quantification. By default, the top matching protein, also known as the “master protein”, was identified. This protein had the largest number of unique peptides and the smallest value in the percent peptide coverage (the longest protein) (Houdelet et al., 2021).

#### 2.4.2 The functional analysis of protein and DEPs

To eliminate errors introduced by the experiment, median normalization was conducted on the original data and filtered out data sets with over 50% null values. The missing data were inputed using the perseus algorithm. Differential expression analysis was performed using criteria for the average ratio-fold change being greater than 1.2 and a *p*-value of less than 0.05.

To analyse various protein families and pathways, COG, KEGG, and Reactome databases were used (Huang et al., 2009; You et al., 2022). The proteome was annotated using the GO database, categorizing proteins based on their molecular function, biological process, and cellular components. Functional descriptions of protein domains were annotated using Pfam, which analysed protein sequence alignments against the Pfam domain database. Enriched pathways were identified and the enrichment of different proteins was evaluated against all identified proteins using the KEGG database and the Hypergeometric distribution. Probable interacting partners were predicted using the STRING database, which contains information about known and predicted protein-protein interactions. Finally, the discriminative ability of candidate biomarkers was assessed using AUC produced by ROC curves.

### 2.5 3D macrophage culture in chiral matrices

To create 3D matrices containing murine RAW264.7 macrophage cell line (Cyagen Biosciences, Inc.), a concentrated gelator solution was first prepared by mixing DMSO with gelator powder until complete dissolution. Then RAW264.7 cells (3 million ml^−1^) suspended in DMEM (Cyagen Biosciences, Inc.) were mixed with the concentrated solution and added into a 48-well plate. The final gelator concentration was 3 mg/mL, with a final DMSO concentration of 3.3% (v/v). Within a few minutes, self-supporting matrices were rapidly formed. After the formation of matrices, additional DMEM was then added, and the matrices were transferred to a humidified incubator under standard culture conditions (37°C, 5% CO_2_).

### 2.6 Immunofluorescence analysis

RAW264.7 cells were cultured in 48-well plates at a density of 1 × 10^6^ cells/mL for 1 day. Following culture, cells were rinsed with phosphate-buffered saline (PBS) and fixed with 4% (w/v) paraformaldehyde at room temperature for 30 min. The samples were then permeabilized with 0.1% (w/v) Triton X-100 (diluted with PBS) for 10 min and blocked with 3% (w/v) bovine serum albumin (BSA; diluted with PBS) for 20 min at room temperature. BSA of 3% (w/v) was then used as blocking solution to minimize nonspecific immunostaining. After removing the permeabilization solution, cells were rinsed with PBS for 5 min at room temperature. Then polyclonal rabbit anti-Akt1 (1:200; Abcam) were added to the samples in 5% (w/v) BSA in PBS and incubated overnight at 4°C. Cells were rinsed thoroughly and then incubated for 1 h in the dark with the secondary antibody goat anti-rabbit IgG H&L Alexa Fluor 488 pre-adsorbed (1:500; Abcam). Phalloidin (Sigma) was used for cytoskeletal staining, and 4’, 6-Diamidino-2-phenylindole (DAPI; Sigma) was used to stain the cell nuclei. A confocal laser scanning microscope (Leica) was utilized to capture images.

### 2.7 Flow cytometry

Cells were isolated by centrifugation at 1,000 rpm for 5 min. Then, 100% methanol was added to fix and permeate the cells. The samples were washed three times with PBS. Primary antibody specific to Akt1 (1:200, Santa Cruz), was added and incubated for 30 min, followed by rinsing with PBS for three times. Subsequently, goat anti-mouse IgG H&L Alexa Fluor 488 fluorescent secondary antibody (1:200, Abcam) was added, incubated for 30 min, and then rinsed with PBS for three times. PE fluorescently labeled primary antibody CD206 (1:200, eBioscience) or Rho (1:200, Abcam) was then added and incubated for 30 min, followed by rinsing with PBS three times. The cell suspension was then prepared using a filter and loaded on the machine. The expression levels of the various protein markers were detected using a flow cytometer, and FlowJo software was utilized for data analysis.

### 2.8 Statistics

Statistical analyses were conducted using SPSS (version 26), and figures were generated using GraphPad Prism (version 9.0). Statistical significance was analyzed with a two-tailed unpaired Student’s t test or analysis of variance (ANOVA) for multiple comparisons.

## 3 Results

### 3.1 Heterogeneity of proteins is affected by matrix chirality *in vivo*

**FIGURE 1:**
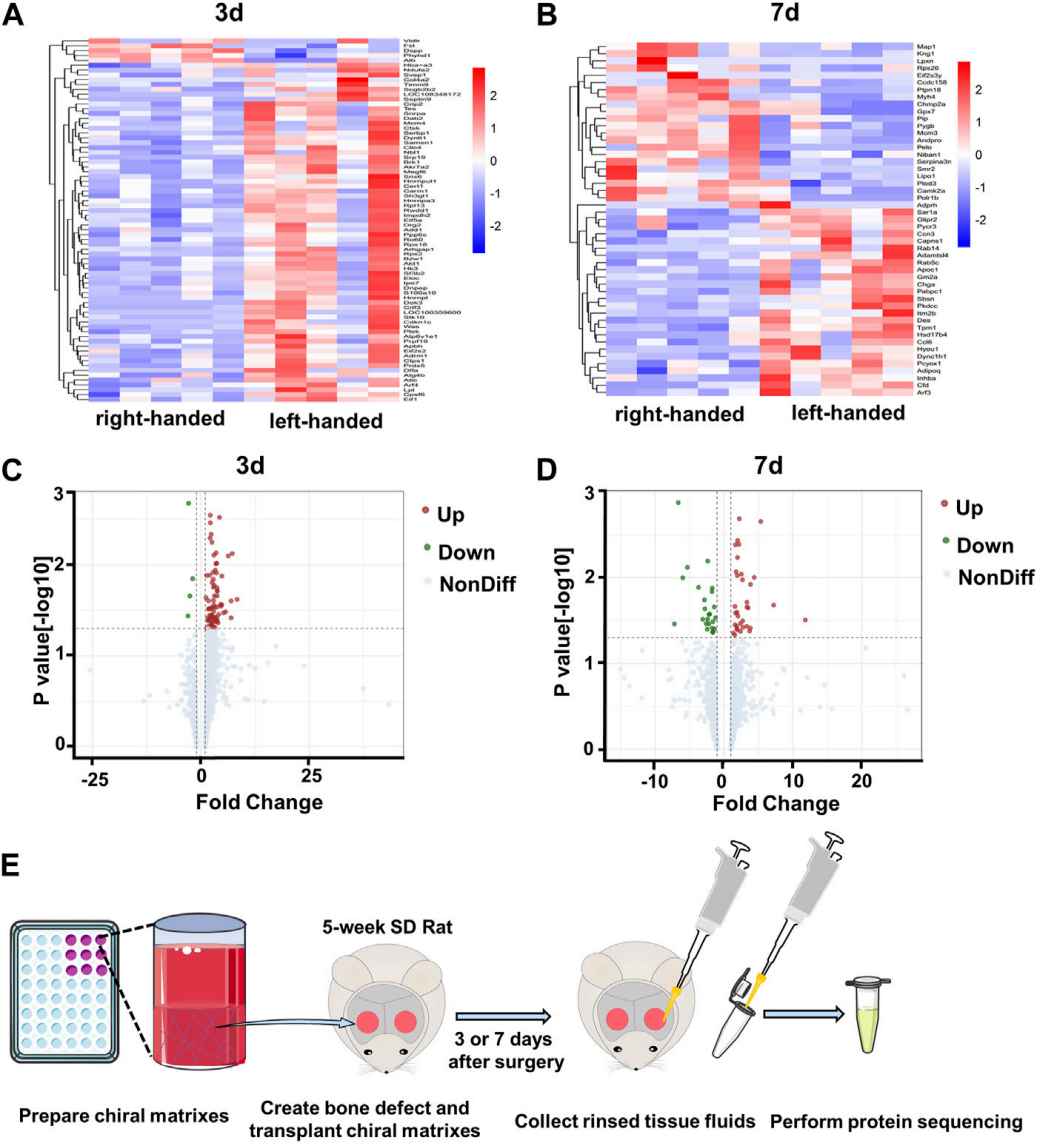
Profiles of proteins collected from bone defect areas transplanted with left-handed and right-handed matrices after 3 days and 7 days. Heatmaps of proteins identified by LC-MS/MS on day 3 **(A)** and day 7 **(B)**. Volcano plots showing differentially-expressed proteins in the presence of left-handed *versus* right-handed matrices on day 3 **(C)** and day 7 **(D)** (*p* < 0.05). **(E)**. Experimental scheme depicting protein collection procedures.

Our previous study revealed that left-handed nanofibrils strongly stimulate M2 polarization of macrophages via mechanotransduction both *in vivo* and *in vitro*, in contrast to right-handed nanofibrils (Jiang et al., 2022). Additionally, left-handed nanofibrils also exerted a favorable impact on bone regeneration (Wei et al., 2019). To better understand the protein components of the bone defect areas biased by chiral matrices, we obtained tissue fluids from the defect regions (Figure 1E), and conducted protein sequencing on 20 samples (2 chiralities × 2 time points × 5 replicates), as shown in Supplementary Figure S1B. A comparison of the left-handed group with the right-handed group revealed a total of 78 differentially expressed proteins (DEPs) on day 3, and 49 DEPs on day 7, with no overlap between the groups (refer to Supplementarys Figures S1C, D). The heatmaps generated for the DEPs on day 3 and day 7 demonstrated clear expression differences between the left-handed and right-handed groups (refer to Figures 1A, B). The volcano plots for identified differentially-expressed proteins on day 3 and day 7 showed both the statistical significance and fold-change of the proteins (refer to Figures 1C, D). On day 3, 74 proteins displayed significantly increased expression, while 4 proteins were downregulated in the left-handed group (Supplementary Table S1). On day 7, 29 proteins were upregulated while 20 were downregulated, with significant changes in expression levels in the left-handed group (Supplementary Table S1). Hence, identified proteins affected by chirality within the bone defect areas can be clearly distinguished.

### 3.2 Left-handed chirality upregulates cell adhesion-related proteins on day 3

**FIGURE 2:**
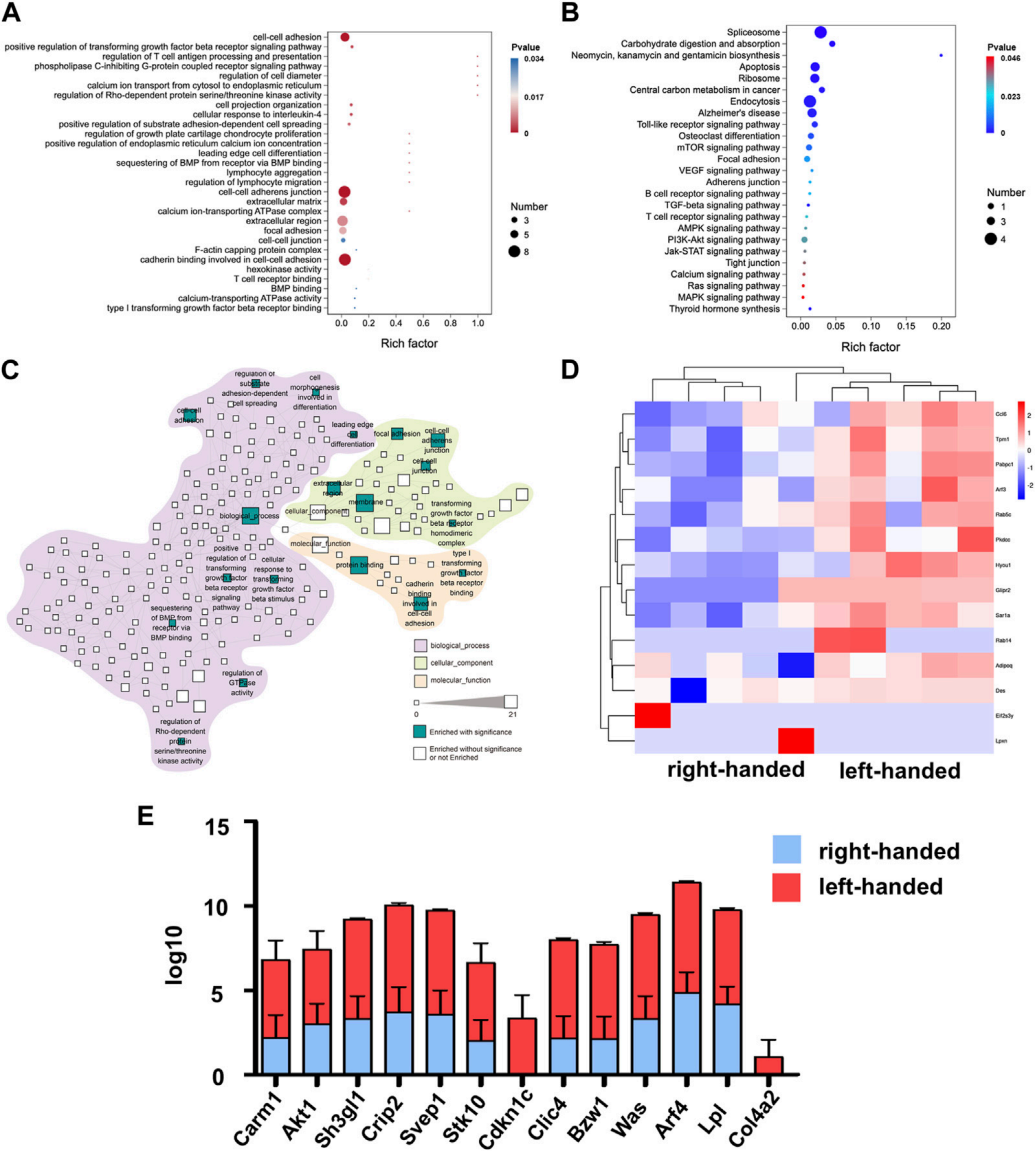
Proteomics analysis of left-handed group-induced cell adhesion on day 3. **(A)** GO enrichment analysis of differential proteomics after 3 days **(B)** KEGG pathway analysis of the DEPs. **(C)** Statistically enriched (*p* < 0.05, calculated with Fisher exact test and Benjamini–Hochberg multiple-testing correction) reactome terms in differentially-expressed proteins. **(D)** Heatmap illustrating DEPs on day 3. **(E)** Relative expression levels of DEGs showed that the left-handed group upregulated cell adhesion-related proteins.

To analyze the specific biological functions of the DEPs on day 3, GO, KEGG and reactome pathway analyses were all performed. The results of the GO analysis revealed that the upregulated proteins in the left-handed group were mainly involved in cell adhesion, including cell-cell adhesion, cell-cell adherent junctions, cell projection organization, leading edge cell differentiation, focal adhesion, and cadherin binding. Additionally, regeneration-related processes were enhanced as well, such as extracellular matrix, positive regulation of transforming growth factor beta receptor signaling pathway, regulation of T-cell antigen processing and presentation, cell response to interleukin-4, regulation of growth plate cartilage chondrocyte proliferation, BMP binding, and type I transforming growth factor beta receptor binding (Figure 2A). The KEGG analysis showed that pathways directly related to mechanotransduction, such as AMPK, PI3K-Akt, Jak-STAT, Ras, and MAPK signaling pathways, were all enriched, confirming the upregulation of cell adhesion pathways in the left-handed group. Consistent with the GO analysis, various regeneration-related pathways such as osteoclast differentiation, VEGF, and T cell receptor signaling pathways were enriched in the KEGG analysis (Figure 2B). The reactome enrichment analysis was used to establish a functional network that linked the identified cell adhesion proteins. Pathways were classified based on their biological processes, cellular components, and molecular functions. The identified pathways were prominent in biological processes, and the signaling pathways with the highest number of identified proteins were related to membrane (21 DEPs) and protein binding (17 DEPs) functions. The enrichment of focal adhesions may be due to previously-established relationships between fibronectin recognition and integrin activation (Jiang et al., 2022), which implies the same initial cell sensing process to chirality. Furthermore, two pathways related to Rho GTPases (regulation of GTPase activity, and regulation of Rho-dependent protein serine/threonine kinase activity) were enriched as well (Figure 2C). These results suggest that Rho GTPases, acknowledged as significant mediators of intracellular mechanotransduction (Xie et al., 2023), could have a function in the transmission of chiral cues. The column and heatmap analyses revealed that the proteins involved in cell adhesion, such as Crip2, Col4a2, Was, Bzw1, Clic4, Lpl, Arf4, Sh3gl1, and Akt1, were significantly upregulated in the left-handed group. Similarly, regeneration-related proteins, such as Cdkn1c, Was, and Carm1, were also upregulated, which was consistent with the GO and KEGG analysis (Figures 2D–F). Our findings thus indicate that the left-hand matrix significantly upregulated cell adhesion and enhanced regeneration on the third day post-transplantation, which is consistent with previous studies (Dou and Feng, 2017).

### 3.3 Left-handed chirality continues to upregulate GTPase-related proteins on day 7

**FIGURE 3:**
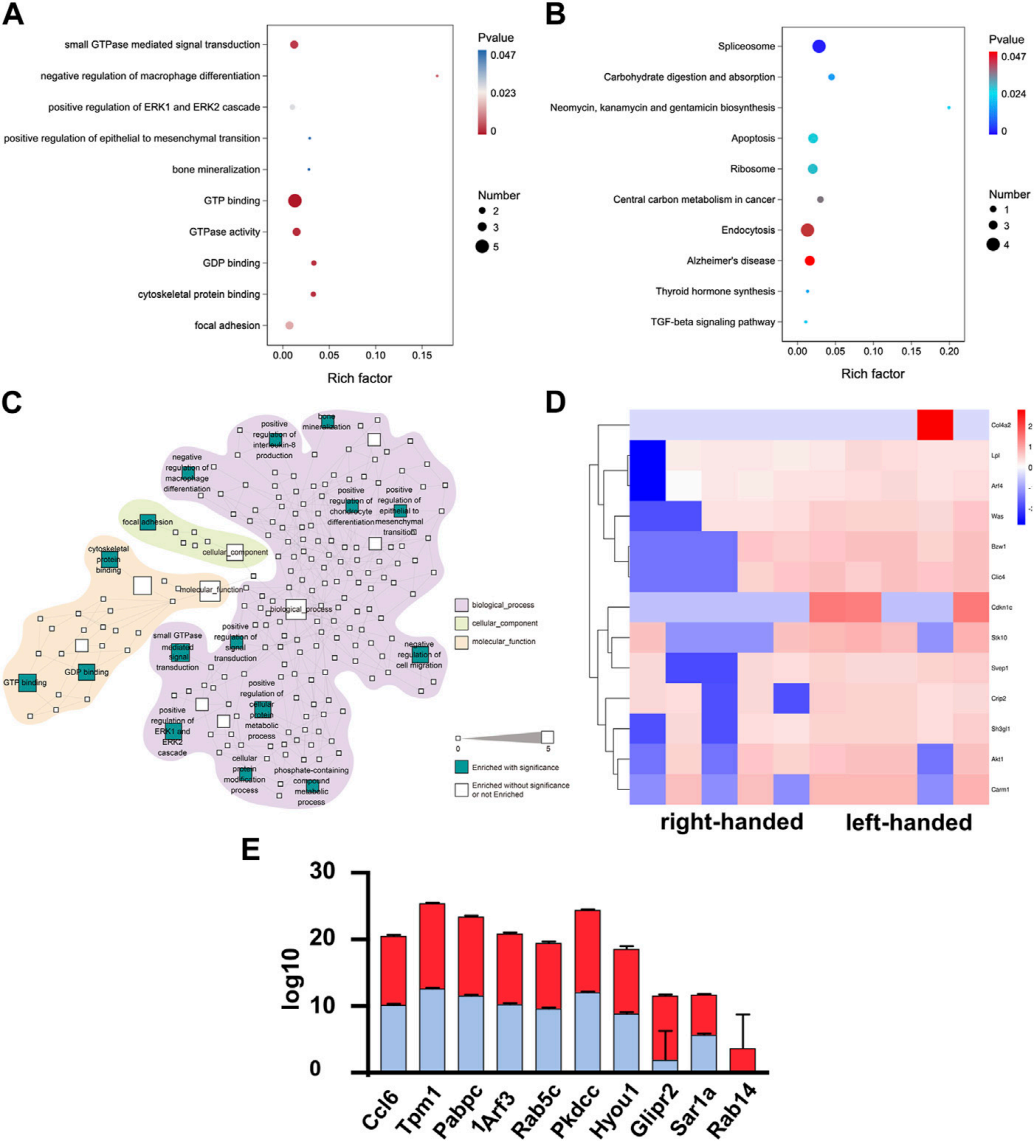
Proteomics analysis demonstrated that the left-handed matrix induced GTPase and regeneration on day 7. **(A)** GO enrichment analysis of differential proteomics after 7 days. **(B)** KEGG pathway analysis of the DEPs. **(C)** Statistically-enriched (*p* < 0.05, calculated with Fisher exact test and Benjamini–Hochberg multiple-testing correction) reactome terms in differentially-expressed proteins. **(D)** Heatmap illustrating DEPs on day 7. Pairwise comparisons are shown for each column. **(E)** Relative expression level of DEPs showing that left-handed matrix upregulated GTPase and regeneration related proteins.

The same methods were used to examine the functions of DEPs on day 7. A decrease in the number of DEPs was observed, as depicted in Supplementary Figure S1C. However, DEPs associated with GTPase remained upregulated in the left-handed group, including proteins involved in GTP binding, GTPase activity, GDP binding, and small GTPase mediated signal transduction, as demonstrated in Figures 3A, C. This observation thus suggests that GTPase may play a crucial role in the regeneration process from day 3 to day 7. Furthermore, the upregulation of matrix remodeling (extracellular matrix glycoproteins and cell adhesion) and mechanotransduction (positive regulation of ERK1 and ERK2 cascade, cytoskeletal protein binding, and focal adhesion) processes in the left-handed group were retained on day 7. Notably, KEGG analysis identified the enrichment of the TGF-beta signaling pathway, which may facilitate tissue repair (Perez et al., 2018). Among the pathways highlighted in Figure 3C, small GTPase mediated signal transduction exhibited the highest number of DEPs. Interestingly, newly-emerged enriched pathways such as bone mineralization, positive regulation of chondrocyte differentiation, epithelial to mesenchymal transition, and negative regulation of cell migration suggested better long-term bone healing processes in the left-handed group. Several GTP and GDP-related proteins, including Rab14, Sar1a, and Rab5c, were upregulated in the left-handed group, reinforcing the upregulation of Rho GTPase pathways (Schwartz et al., 2008; Barbera et al., 2019). The expression levels of cell adhesion-related proteins, such as Ccl6, Tpm1 and Pabpc1, were also higher in the left-handed group, as depicted in Figures 3D–F. Overall, these observations highlight the significant upregulation of GTPase-related pathways during the early phase of bone regeneration.

### 3.4 Akt1 and GTPase activation play a central role in protein heterogeneity

**FIGURE 4:**
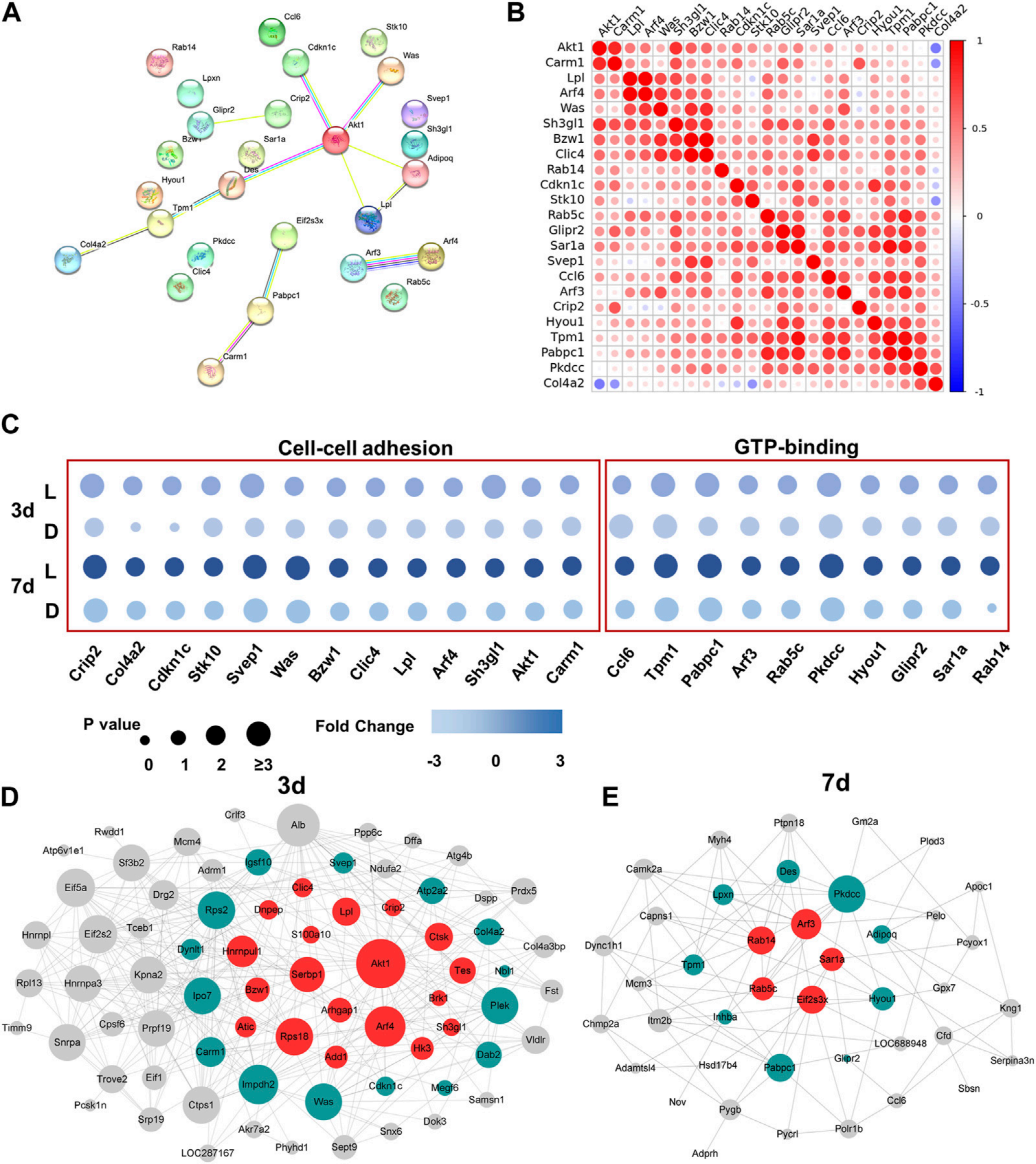
Akt1 and GTPase activation played a key role in protein heterogeneity between day 3 and day 7. **(A)** PPI networks of DEPs from 3 days to 7 days. Each node represents the relevant protein; with line thickness indicating the strength of the supporting data. **(B)** Correlation analysis of DEPs. Non-significant correlations (<0.05 adjusted *p*-value) are shown in white; colored bar indicates Pearson’s r value. **(C)** Independent validation of cell-cell adhesion and GTP-binding related proteins. Construction of the PPI network on day 3 **(D)** and day 7 **(E)**.

To investigate the interactions of DEPs between day 3 and day 7, we employed the STRING tool to analyze protein-protein interaction (PPI) relationships for the target proteins. From the 127 DEPs, we selected 23 DEPs that exhibited the greatest significance for further analysis. The results revealed that Akt1 played a central role in protein-protein analysis among all the DEPs from day 3 and day 7, highlighting its importance in the chirality-biased regeneration process (as illustrated in Figure 4A). The pairwise protein correlation heatmap demonstrated that most adhesion proteins were positively correlated with each other and with Akt1, further validating its central role (as shown in Figure 4B). The bubble plot illustrated that the screened 23 DEPs involved in cell-cell adhesion and GTP-binding signaling were mostly upregulated in the left-handed group (Figure 4C). Construction of the PPI networks separately for day 3 and day 7 showed that Akt1 and other adhesion proteins were situated within the central nodes of the network on day 3, whereas GTPase-related proteins were located at the center on day 7 (as seen in Figures 4D, E). As such, Akt1 and Rho GTPase were the crucial and central components of protein interactions on day 3 and day 7, respectively.

### 3.5 Akt1 and Rho GTPase are essential for macrophage polarization to the M2 phenotype

**FIGURE 5:**
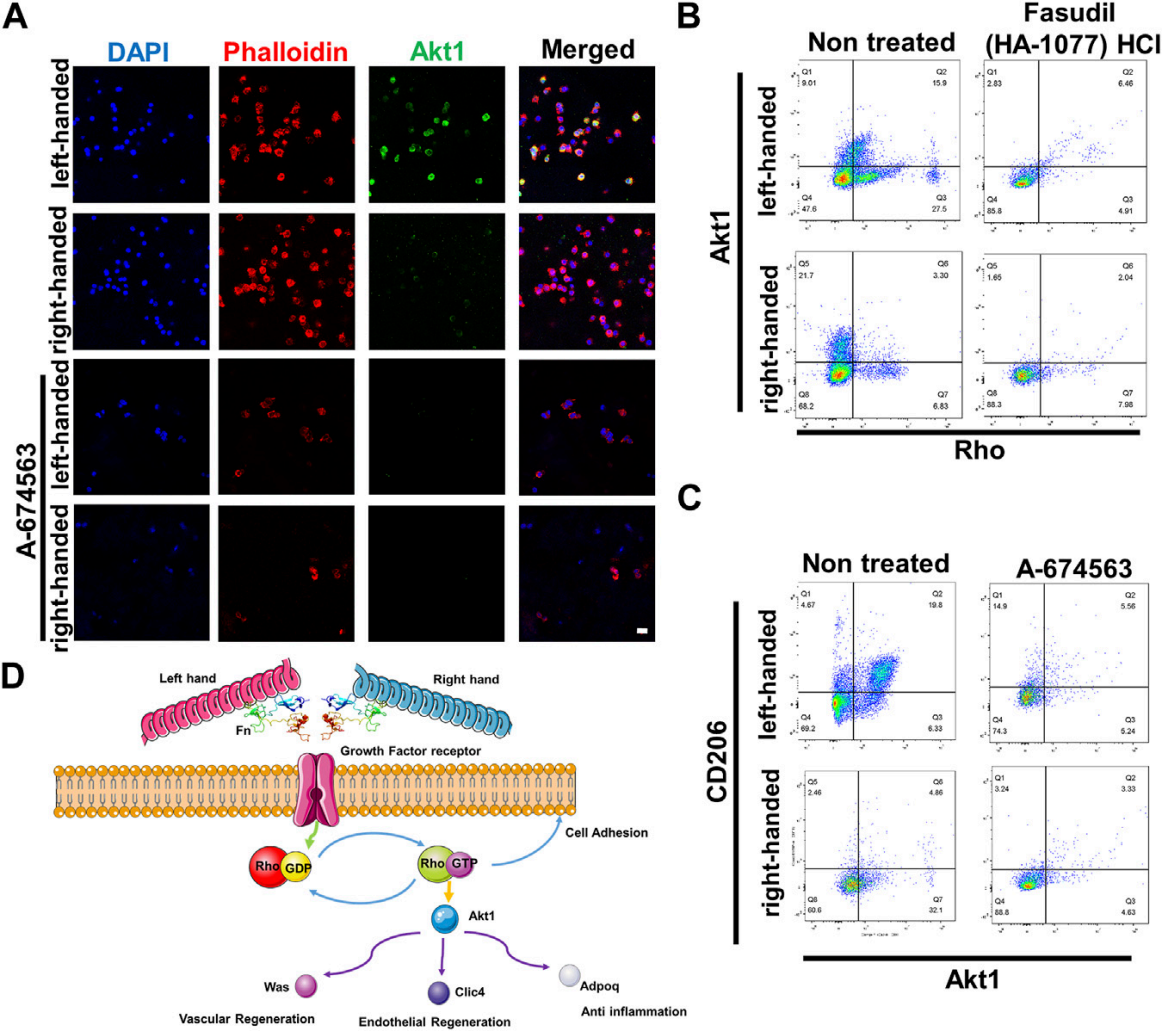
Akt1 and GTPase activation are essential for macrophage polarization to the M2 phenotype. **(A)** Immunofluorescence images showed that inhibition of Akt1 greatly reduced the M2 polarization of macrophages in chiral matrices (scale bar: 10 μm). **(B)** Flow cytometry showed that Rho inhibition suppressed the expression of Rho and downstream Akt1. **(C)** Flow cytometry showed that Akt1 inhibition suppressed the expression of Akt1 and downstream CD206. **(D)** Schematic representation of chirality-dependent immunological responses and downstream effects on tissue regeneration mediated by Akt1.

To validate protein sequencing results of the functions of Akt1 and Rho GTPase in the regeneration process, we cultured macrophages (RAW 264.7) in chiral matrices. Results from immunofluorescence staining indicated that Akt1 is significantly upregulated in the left-handed group, and inhibiting Akt1 using A-674563 downregulated Akt1 expression in both the left-handed and right-handed groups (Figure 5A). Additionally, data obtained from flow cytometry testing indicated that inhibiting Rho using Fasudil (HA-1077) HCl greatly suppressed downstream Akt1 expression (Figure 5B). Furthermore, inhibiting Akt1 led to a reduction in the expression of CD206, the macrophage M2 marker (Figure 5C). Besides, downstream Was, Clic4, Adipoq and Rab showed a positive effect on tissue regeneration (Jiang et al., 2010; Lovren et al., 2010; Dewidar et al., 2015; Warner et al., 2019). Hence, we could conclude that both Rho and Akt1 played a crucial role in chirality-mediated macrophage polarization, which in result impacted the regeneration process (Figure 5D).

## 4 Discussion

Understanding the effects of chirality on cell and tissue regeneration is of key importance for the design and application of chiral biomaterials. While previous research has explored its effects using *in vivo* and *in vitro* validations, as well as RNA sequencing (Zhang et al., 2022), little is known about its proteomic characteristics. To address this gap in knowledge, we established an *in vivo* bone defect model and conducted protein sequencing at the 3-day and 7-day time points to investigate how chirality affects temporal regulation of proteins during the early stages of bone regeneration. Our findings revealed, for the first time, that chirality could cause varying activation of Rho GTPase and downstream Akt1, ultimately leading to heterogeneity in macrophage polarization, which could thus explain the disparity in bone repair outcomes.

Properly reconstructing injured bone tissues requires timely activation of the immune response and an appropriate microenvironment to facilitate healing. Cells of the innate immune system are able to sense the microenvironment and react accordingly (Sutherland et al., 2023). Previous research has shown that physical cues such as biomaterials can significantly influence macrophage behavior and regulate macrophage adhesion (Kartikasari et al., 2021; Zhao et al., 2023). For example, suitable diameter of TiO_2_ were found to adsorb fibronectin and vitronectin, regulate M2 polarization of macrophages and inhibit the expression of inflammatory cytokines (Lu et al., 2015). Besides, magnetic fields with low oscillation frequency can promote M2 polarization by activating cell adhesion, while high oscillation frequency promotes M1 polarization (Kang et al., 2017). Chirality can also act as an important cue in regulating macrophage adhesion and polarization as demonstrated in our previous study (Jiang et al., 2022). Specifically, left-handed chirality can significantly inhibit inflammation and promote tissue regeneration by differentially initiating cell sensing and activating mechanotransduction signaling. However, further research is necessary to fully understand how this mechanism regulates tissue regeneration *in vivo*. Through proteomic analysis, our work showed a heterogeneity of proteins within bone defect areas implanted with different chiral matrices. We found that a left-handed matrix can significantly upregulate the expression of adhesion-related proteins, which supports tissue regeneration in the bone defect area.

Downstream mechanotransduction plays key roles in various processes during tissue regeneration as well. The Rho family of GTPases can receive and transduce various cellular signaling cues and further lead to actin nucleation (Ridley, 2006). Injury-responsive Rho GTPases can drive cytoskeleton polymerization and projection retraction of stem cells, which enables them to break the static state, react to injury and induce tissue remodeling (Jiang et al., 2021; Kann et al., 2022). Furthermore, on smaller honeycomb structures of TiO_2_ surface topography, the up-regulation of the Rho family of GTPases has been reported to activate M2 macrophage polarization-related signaling pathways (Aflaki et al., 2011; Zhu et al., 2021). Akt is one of the downstream protein family activated by Rho GTPase pathways (Weichhart et al., 2015). Although sharing common upstream activators, Akt1, different from Akt2 and Akt3, was reported to specifically exert a positive effect on M2 polarization of macrophages (Arranz et al., 2012; Vergadi et al., 2017; Linton et al., 2019). In our study, Rho GTPase and Akt1 were identified as key proteins in regulating the macrophage response affected by chirality through proteomic analysis and *in vitro* verification.

Taken together, our study demonstrated the formation of a protein network driven by chirality *in vivo,* which served as a unique feature during early stages of bone regeneration. More importantly, we found that compared with right-handed chirality, the network of left-handed chirality affects macrophage polarization through the activation of Rho GTPase and Akt1, and further assist the regeneration process. Our findings may enhance the comprehension of immunological responses during tissue regeneration, as well as enabling better understanding of the mechanisms of chirality-mediated regeneration processes.

## 5 Conclusion

In summary, we constructed an atlas of chirality-biased proteins during the early stages of bone tissue regeneration *in vivo*, and uncovered the patterns of DEPs and protein interaction networks. Moreover, we explored the underlying molecular mechanisms of differences in chiral-mediated macrophage polarization. Our results showed that left-handed matrices significantly upregulated the expression of Rho GTPase and Akt1, and drived M2 polarization of macrophages. Our findings provide further insight into the complex interplay between chiral materials and tissue regeneration.

## Data Availability

The original contributions presented in the study are publicly available. This data can be found here: https://proteomecentral.proteomexchange.org/cgi/GetDataset?ID=PXD043696, accession number PXD043696, https://www.iprox.cn/page/project.html?id=IPX0006714000.
