# Supplementary material for: Chirality-biased protein expression profile during early stages of bone regeneration
Source: Front Bioeng Biotechnol. 2023 Jul 18;11:1217919. doi: 10.3389/fbioe.2023.1217919 (PMC10393040; doi:10.3389/fbioe.2023.1217919)
Supplement: Supplementary file 1 [file DataSheet1.docx]

**Supporting Information**


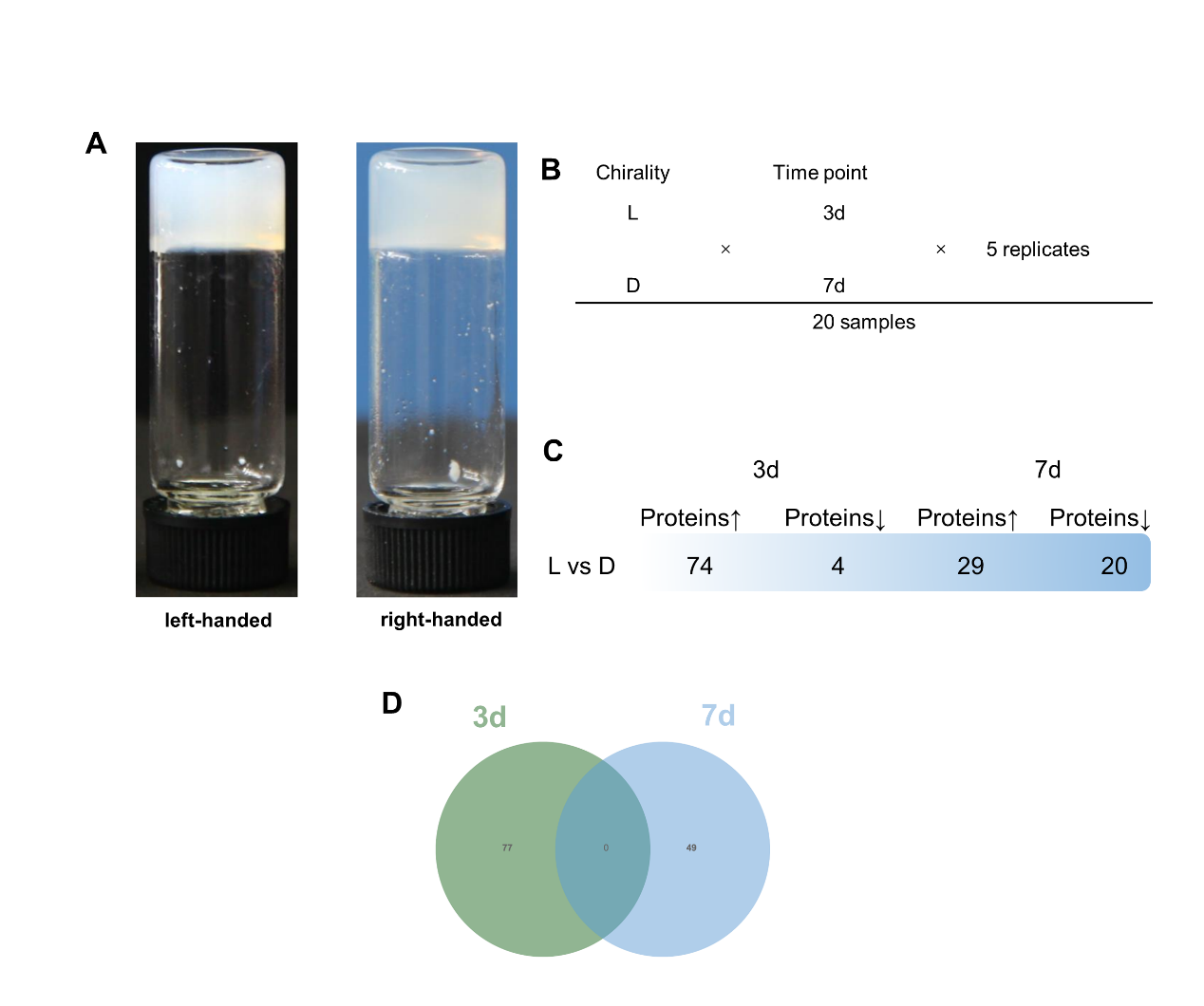


**Figure SI** 1 (A) Chiral matrices used in the experiments. (B) Scheme showing 4 different and 20 samples (2 chirality matrixes ×2 timepoints ×5 replicates) assessed by protein sequencing. (C) Scheme indicating the number of differentially expressed proteins in bone defect areas after transplant with chiral matrix on 3d and 7d (FDR<0.05). (D) Venn-diagram showing the overlap between differentially expressed proteins in bone defect areas after transplant with chiral matrixes after 3d and 7d (FDR<0.05).

**Table SI 1** DEPs on day 3.

| **Gene Name** | **FC** | **pValue** |
| --- | --- | --- |
| Col4a2 | -2.3573842 | 0.00461 |
| Cdkn1c | -4.540893 | 0.027258 |
| Akt1 | -3.7693888 | 0.019384 |
| Ctsk | -2.6099916 | 0.015658 |
| Clic4 | -3.4535992 | 0.009723 |
| Was | -7.2463768 | 0.007504 |
| Carm1 | -3.3485357 | 0.049179 |
| Snrpa | -3.5935546 | 0.037891 |
| Hnrnpul1 | -3.9515383 | 0.043381 |
| Srp19 | -3.3081801 | 0.011472 |
| LOC108348172 | -6.96767 | 0.024889 |

**Table SI 1** (continued)

| **Gene Name** | **FC** | **pValue** |
| --- | --- | --- |
| Eif1 | -3.7265322 | 0.021866 |
| LOC100359600 | -3.596566 | 0.012272 |
| Hnrnpl | -6.9785619 | 0.038157 |
| Ppp6c | -2.5272565 | 0.048306 |
| Cpsf6 | -2.1760891 | 0.003441 |
| Sh3gl1 | -3.6150676 | 0.009633 |
| Igsf10 | -1.9150925 | 0.030162 |
| Dok3 | -2.4199774 | 0.043137 |
| Crlf3 | -6.3158427 | 0.007968 |
| Bzw1 | -3.7221352 | 0.029004 |
| Scgb2b2 | -4.1127553 | 0.035078 |
| Ndufa2 | -2.9137274 | 0.017581 |
| Hba-a3 | -2.1384519 | 0.030291 |
| Septin9 | -4.8146133 | 0.027384 |
| Lpl | -2.9917279 | 0.02747 |
| Arf4 | -2.8583924 | 0.014414 |
| Dffa | -4.84156 | 0.013269 |
| Atp2a2 | -3.4984852 | 0.00739 |
| Atic | -2.2931993 | 0.02976 |
| Hk3 | -2.3943952 | 0.035404 |
| Septin9 | -2.7276348 | 0.036981 |
| Megf6 | -4.2859225 | 0.003008 |
| Tes | -4.6565991 | 0.028308 |
| Eloc | -3.5380196 | 0.040247 |
| Atp6v1e1 | -5.704051 | 0.032851 |
| Adrm1 | -3.7855702 | 0.019245 |
| Prpf19 | -3.0655193 | 0.041991 |
| Cert1 | -3.6339586 | 0.009661 |
| Plek | -2.1203421 | 0.005069 |
| Ipo7 | -3.061137 | 0.038286 |
| Rwdd1 | -2.6368596 | 0.029372 |
| Snx6 | -2.4864427 | 0.043652 |
| Sf3b2 | -5.2335196 | 0.033528 |
| Nbl1 | -2.0228297 | 0.013013 |
| Rps18 | -2.25312 | 0.04603 |
| Prdx5 | -3.7120903 | 0.017792 |
| Stk10 | -8.3461307 | 0.02387 |
| Dnpep | -2.4621748 | 0.048573 |
| Akr7a2 | -3.2392546 | 0.037205 |
| Timm9 | -3.1267197 | 0.021914 |
| Crip2 | -3.0124474 | 0.047799 |
| Add1 | -1.6990361 | 0.047506 |

**Table SI 1** (continued)

| **Gene Name** | **FC** | **pValue** |
| --- | --- | --- |
| Eif5a | -2.2578052 | 0.037079 |
| Drg2 | -2.1802975 | 0.038018 |
| Apbh | -3.7764208 | 0.040427 |
| Ro60 | -1.6524774 | 0.041318 |
| Dynlt1 | -2.9029599 | 0.024517 |
| Rps2 | -2.0637662 | 0.035796 |
| Svep1 | -2.7304351 | 0.038648 |
| Hnrnpa3 | -2.0355903 | 0.039443 |
| Ctps1 | -3.1136934 | 0.029433 |
| S100a10 | -1.73123 | 0.030654 |
| Dab2 | -2.6993468 | 0.047738 |
| Brk1 | -2.5573289 | 0.005645 |
| Samsn1 | -2.1615593 | 0.002842 |
| Arhgap1 | -2.3233823 | 0.046615 |
| Impdh2 | -1.8329353 | 0.040565 |
| Rpl13 | -1.8658596 | 0.040158 |
| Atg4b | -2.4978706 | 0.03573 |
| Serbp1 | -3.4312615 | 0.041108 |
| Eif2s2 | -1.53957 | 0.03804 |
| Alb | 2.96235 | 0.036459 |
| Vldlr | -1.7706636 | 0.02486 |
| Mcm4 | -2.9352886 | 0.032519 |
| Phyhd1 | 1.904049 | 0.014195 |
| Dspp | 2.845362 | 0.002103 |
| Fst | 2.526072 | 0.021957 |

**Table SI 2** DEPs on day 7.

| **Gene Name** | **FC** | **pValue** |
| --- | --- | --- |
| Chga | -11.808606 | 0.031129 |
| Inhba | -3.3068892 | 0.036922 |
| Itm2b | -2.7548665 | 0.037913 |
| Pelo | 2.834065 | 0.018157 |
| Pcyox1 | -2.5182764 | 0.009134 |
| Rab14 | -2.4038866 | 0.042305 |
| Adprh | -1.9665606 | 0.005829 |
| Gpx7 | 2.59244 | 0.034289 |
| Hsd17b4 | -3.8615704 | 0.011987 |
| Lpxn | -2.6988441 | 0.010595 |
| Arf3 | -2.0365272 | 0.028051 |
| Adamtsl4 | -1.9374438 | 0.009457 |
| Sar1a | -2.2274245 | 0.002065 |
| Capns1 | -1.897562 | 0.025301 |

**Table SI 2** (continued)

| **Gene Name** | **FC** | **pValue** |
| --- | --- | --- |
| Myh4 | 3.693103 | 0.012965 |
| Andpro | 6.634167 | 0.001335 |
| Ccn3 | -1.9605652 | 0.040476 |
| Ptpn18 | 5.305587 | 0.007597 |
| Rab5c | -1.8570654 | 0.039474 |
| Des | -5.342394 | 0.002223 |
| Lipo1 | 2.177131 | 0.034449 |
| Mcm3 | 2.354975 | 0.006432 |
| Cfd | -4.3960295 | 0.009951 |
| Pip | 2.379456 | 0.030433 |
| Adipoq | -1.6230341 | 0.035429 |
| Niban1 | 2.067146 | 0.026535 |
| Tpm1 | -1.6695327 | 0.004138 |
| Hyou1 | -7.2186009 | 0.020879 |
| Sbsn | -2.727672 | 0.031898 |
| Pabpc1 | -2.1810584 | 0.004091 |
| Plod3 | 1.606781 | 0.032454 |
| Chmp2a | 2.428958 | 0.039873 |
| Apoc1 | -2.3780817 | 0.034173 |
| Dync1h1 | -3.3365698 | 0.019287 |
| Serpina3n | 1.693299 | 0.01321 |
| Pkdcc | -1.6867246 | 0.025752 |
| Pycr3 | -1.8521537 | 0.036872 |
| Pygb | 3.077494 | 0.030501 |
| Camk2a | 2.766489 | 0.023076 |
| Rps26 | 1.711953 | 0.038657 |
| Glipr2 | -3.5102006 | 0.022426 |
| Smr2 | 7.193154 | 0.034506 |
| Ccdc158 | 1.649703 | 0.043134 |
| Map1 | 1.588257 | 0.043281 |
| Gm2a | -3.3373938 | 0.021994 |
| Ccl6 | -1.5661683 | 0.046349 |
| Eif2s3y | -1.7632766 | 0.008534 |
| Kng1 | 1.742588 | 0.014534 |
| Polr1b | 5.976616 | 0.010047 |
